# How many people in the Netherlands live with a hip, knee, or shoulder replacement? prevalence estimates using data from the Dutch Arthroplasty Register (LROI) and Statistics Netherlands

**DOI:** 10.1302/2633-1462.61.BJO-2024-0162.R1

**Published:** 2025-01-13

**Authors:** Mirthe H. W. van Veghel, Liza N. van Steenbergen, Maaike G. J. Gademan, Wilbert B. van den Hout, B. W. Schreurs, Gerjon Hannink

**Affiliations:** 1 Department of Orthopaedics, Radboud University Medical Center, Nijmegen, The Netherlands; 2 Dutch Arthroplasty Register (Landelijke Registratie Orthopedische Interventies), ‘s-Hertogenbosch, The Netherlands; 3 Department of Orthopaedics, Leiden University Medical Center, Leiden, The Netherlands; 4 Department of Clinical Epidemiology, Leiden University Medical Center, Leiden, The Netherlands; 5 Department of Biomedical Data Sciences, Leiden University Medical Center, Leiden, The Netherlands; 6 Department of Medical Imaging, Radboud University Medical Center, Nijmegen, The Netherlands

**Keywords:** Prevalence, Joint replacement, Poisson regression analyses, Parametric survival models, Arthroplasty registry, shoulder arthroplasties, knee, hip, joint replacements, knee arthroplasty procedures, hip and knee arthroplasties, shoulder, Dutch Arthroplasty Register (LROI), regression analyses, primary arthroplasties

## Abstract

**Aims:**

We estimated the prevalence of people living with at least one hip, knee, or shoulder arthroplasty in the Netherlands.

**Methods:**

We included the first hip (n = 416,333), knee (n = 314,569), or shoulder (n = 23,751) arthroplasty of each patient aged ≥ 40 years between 2007 and 2022 (hip/knee) or 2014 and 2022 (shoulder) from the Dutch Arthroplasty Register (LROI). Data on the size of the Dutch population were obtained from Statistics Netherlands. Annual incidences and deaths from hip and knee arthroplasty since 2010, and shoulder arthroplasty since 2015, were observed from the LROI. Annual incidences and deaths before those years were estimated using Poisson regression analyses and parametric survival models based on a Gompertz distribution. Non-parametric percentile bootstrapping with resampling was used to estimate 95% CIs.

**Results:**

Annual incidences per 100,000 Dutch inhabitants aged ≥ 40 years increased for hip arthroplasties from 221 (95% CI 214 to 229) in 1990 to 360 in 2022, for knee arthroplasties from 181 (95% CI 174 to 188) to 272, and for shoulder arthroplasties from 11 (95% CI 8.0 to 16) to 34. In 2022, 791,000 (95% CI 787,000 to 794,000) people in the Netherlands were living with at least one joint replacement, representing 8.4% (95% CI 8.4 to 8.5) of the Dutch population aged ≥ 40 years. For hip, knee, and shoulder arthroplasties, these were 436,000 (95% CI 433,000 to 438,000), 383,000 (95% CI 380,000 to 386,000), and 34,000 (95% CI 33,000 to 36,000) people, corresponding to 4.7% (95% CI 4.6 to 4.7), 4.1% (95% CI 4.1 to 4.1), and 0.4% (95% CI 0.3 to 0.4) of the Dutch population, respectively. The most common age group living with at least one joint replacement was the ≥ 80-year age group, representing 38% (95% CI 37 to 38) of the Dutch population aged ≥ 80 years.

**Conclusion:**

Approximately 800,000 people in the Netherlands were living with at least one hip, knee, or shoulder replacement in 2022, representing one in 12 Dutch inhabitants aged ≥ 40 years.

Cite this article: *Bone Jt Open* 2025;6(1):74–81.

## Introduction

In recent decades, there has been an increase in the number of arthroplasties in the Netherlands and worldwide.^1^ There, the annual number of hip and knee arthroplasties increased from 50,000 in 2010 to 80,000 in 2022. For shoulder arthroplasties, this doubled from 2,000 in 2014 to 4,000 in 2022. Due to the ageing population and the increasing use of arthroplasties in younger patients and vulnerable elderly patients, annual procedure volumes could increase even further in the coming years.^2-4^

Since 2007, the Dutch Arthroplasty Register (LROI) has been registering hip and knee arthroplasties in the Netherlands.^5^ In 2014, shoulder arthroplasties were added to the LROI database. Registry data can be used to estimate the prevalence of people living with a joint replacement. This provides insight into the number of individuals at risk for associated complications, such as periprosthetic joint infections (PJIs) or periprosthetic fractures, which may lead to higher mortality and higher revision rates.^6-9^ In addition, the prevalence of people living with a joint replacement can be used to project future demand for primary and revision arthroplasty, which is crucial for policy-makers in government, education, and industry.^10-12^

To our knowledge, the prevalence of people with a joint replacement in the Netherlands has not been estimated before. Therefore, this study aimed to estimate the prevalence of people living with at least one joint replacement in the Netherlands stratified by joint, age, and sex, using data from the LROI and Statistics Netherlands (Centraal Bureau voor de Statistiek (CBS)).

## Methods

Patient-level data were obtained from the LROI, which is the national population-based arthroplasty register of the Netherlands, established by the Netherlands Orthopaedic Association (NOV). In 2012, 100% coverage of Dutch hospitals was achieved with a completeness of more than 95% of primary hip and knee arthroplasties.^5^ Nowadays, completeness of primary hip, knee, and shoulder arthroplasties is reported to be higher than 97%.^1^ The LROI contains data on patient, prosthesis, and procedure characteristics of primary and revision arthroplasties. The vital status of all patients is obtained at regular time intervals from Vektis (Zeist, the Netherlands), which is the national insurance database on healthcare in the Netherlands, recording all deaths of Dutch inhabitants.

**Fig. 1:**
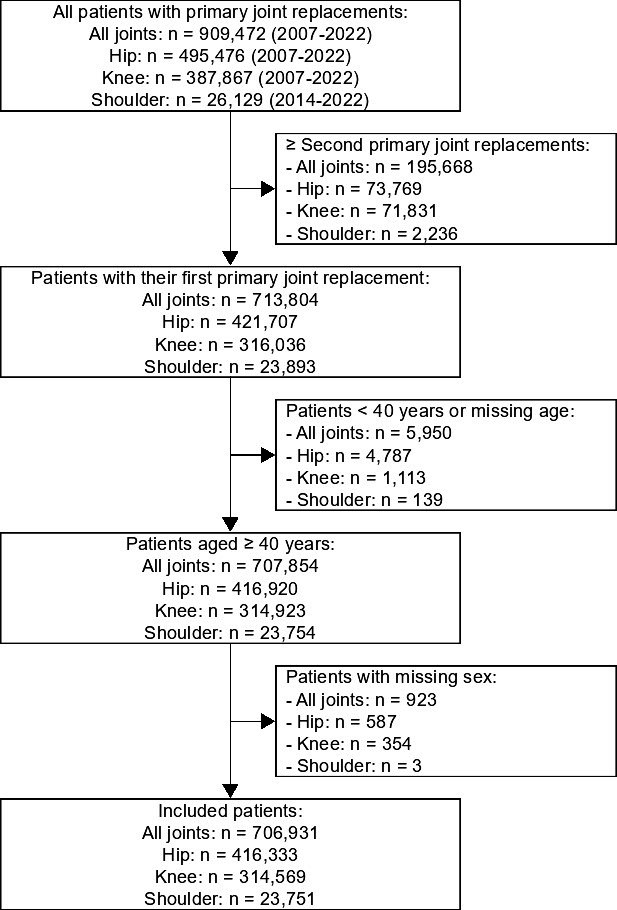
Flowchart.

In this study, we included all patients aged ≥ 40 years with their first primary hip, knee, or shoulder arthroplasty (n = 706,931) registered in the LROI between 2007 and 2022 (Figure 1). In addition to the entire cohort, separate cohorts were identified for each joint to present joint-specific results (i.e. first primary hip (n = 416,333) or first primary knee (n = 314,569) arthroplasty registered in the LROI between 2007 and 2022, and first primary shoulder (n = 23,751) arthroplasty registered between 2014 and 2022). Patients aged ≥ 40 years comprise more than 98% of all first primary hip, knee, and shoulder arthroplasties in the Netherlands. Patients with missing sex data (no female/male) were excluded (all joints n = 923; hip n = 587; knee n = 354; shoulder n = 3). The sum of the joint-specific cohorts included more patients than the entire cohort, as the entire cohort included only the first primary hip, knee, or shoulder arthroplasty for each patient. Patients with primary arthroplasties in multiple joints could be included in multiple joint-specific cohorts. Publicly available data on the size of the Dutch population were obtained from CBS for the period 1990 to 2022, categorized by calendar year, age (years), and sex (female/male). Dutch people aged ≥ 99 years were classified as 99-year-olds, as CBS grouped all individuals aged ≥ 99 years into a single age category until 1995. This study was reported in accordance with the STROBE guidelines.

### Ethics

Data were available from the LROI, however restrictions apply to the availability of these data, which were used under licence for the current study. All data were received completely de-identified. The LROI uses the opt-out system to require informed consent from patients.

### Statistical analysis

The annual incidences of primary hip and knee arthroplasties since 2010, and of primary shoulder arthroplasties since 2015, were observed from the LROI, as the LROI has been nearly complete for these arthroplasties since 2010 and 2015, respectively.^1^ Incidence was calculated as the number of new primary hip, knee, or shoulder arthroplasties per year divided by the total number of Dutch inhabitants aged ≥ 40 years. Since there are patients alive with a primary hip, knee, or shoulder replacement before those years, the annual incidences of primary hip and knee arthroplasties before 2010 and of primary shoulder arthroplasties before 2015 were estimated using Poisson regression analyses. Data from 2010 to 2019 (hip and knee) or from 2015 to 2019 (shoulder) were used to exclude the first incomplete LROI years and the COVID-19 pandemic, where the annual number of arthroplasties was considerably lower than expected.^1,13^ The annual incidences of primary hip, knee, and shoulder arthroplasties were estimated back to 1990. Calendar year (1990 to 2022), sex (female/male), and age (≥ 40 years, continuous) were included as covariates in the Poisson regression models to account for differences in incidence between subgroups and in the general population as well as the changes over time.

The annual number of deaths of patients who had their joint replacement since 2010 (hip and knee) or since 2015 (shoulder) was observed from the LROI to obtain the number of living patients since 2010 or 2015, respectively. Parametric survival models based on a Gompertz distribution were used to estimate the survival probability of patients who had their joint replacement before those years, using LROI data from 2007 to 2022 (hip and knee) and from 2014 to 2022 (shoulder). As individuals are observed only if their event of interest takes place after some specified age (i.e. age at LROI entry), both right and left truncation occurs and was taken into account. The reference age was taken as the age at LROI entry, and survival was calculated from that reference age on. Therefore, conditional survival was used, which was defined as the time from the patient’s age at surgery to the patient’s age at death or end of follow-up (1 January 2023). Survival curves were stratified by an interaction term between sex and procedure year (first five procedure years/later years). The first five procedure years were used for predicting the survival of patients who had their arthroplasty before 2010 (hip and knee) or 2015 (shoulder), as both healthier, younger patients and more vulnerable elderly patients now undergo arthroplasties, whereas they may not have been eligible for these procedures in the past.

Subsequently, the estimated arthroplasties from before the start of the LROI were combined with the observed (i.e. registered) arthroplasties. The combined dataset included all registered cases with a weight of 1. In addition, all unique combinations of pre-registration calendar year, sex, and age were added to the dataset, with a weight equal to the number of arthroplasties as predicted by the Poisson regression. Patient survival status of these pre-registration cases was set to the probability to survive until the specific calendar year, as predicted by the survival analysis. Prevalence was then estimated by the weighted sum of the survival status. Non-parametric percentile bootstrapping of the combined Poisson regression and survival analysis were used to calculate 95% CIs for the prevalence.^14^ A total of 500 bootstrap samples were generated.

All analyses were performed for the entire cohort, as well as for the joint-specific cohorts. Reported numbers greater than or equal to 10,000 were rounded to the nearest thousand, numbers between 1,000 and 9,999 were rounded to the nearest hundred, and numbers less than 1,000 were rounded to the nearest ten. R v. 4.3.2 (R Foundation for Statistical Computing, Austria) was used to perform all analyses.

## Results

**Fig. 2:**
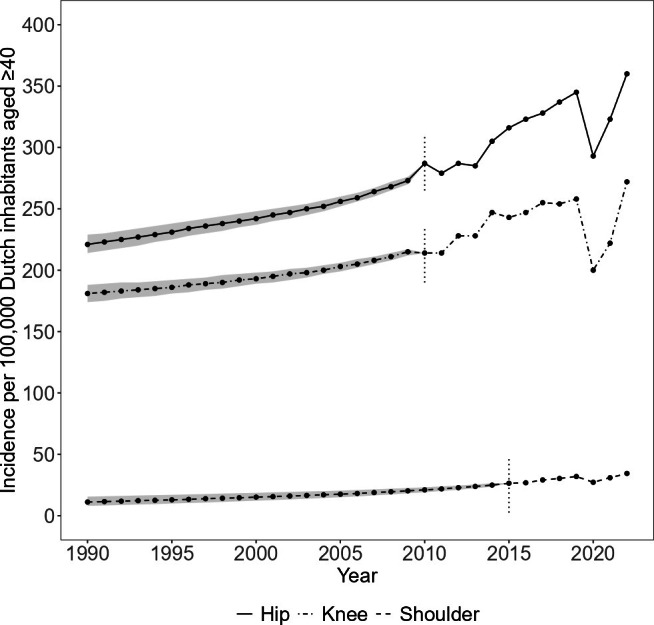
Annual incidence of primary arthroplasties per 100,000 Dutch inhabitants aged ≥ 40 years, stratified by joint. Annual incidences before 2010 (hip and knee) and before 2015 (shoulder) are estimated.

The annual incidences per 100,000 Dutch inhabitants aged ≥ 40 years increased for primary hip arthroplasty from 221 (95% CI 214 to 229) in 1990 to 360 in 2022, for primary knee arthroplasty from 181 (95% CI 174 to 188) in 1990 to 272 in 2022, and for primary shoulder arthroplasty from 11 (95% CI 8.0 to 16) in 1990 to 34 in 2022 (Figure 2). At the start of the LROI in 2007, the incidence per 100,000 Dutch inhabitants of hip and knee arthroplasties was estimated at 264 (95% CI 260 to 267) and 208 (95% CI 205 to 211), respectively. The estimated incidence of primary shoulder arthroplasties was 25 (95% CI 24 to 26) per 100,000 Dutch inhabitants when shoulder arthroplasties were added to the LROI in 2014.

**Fig. 3:**
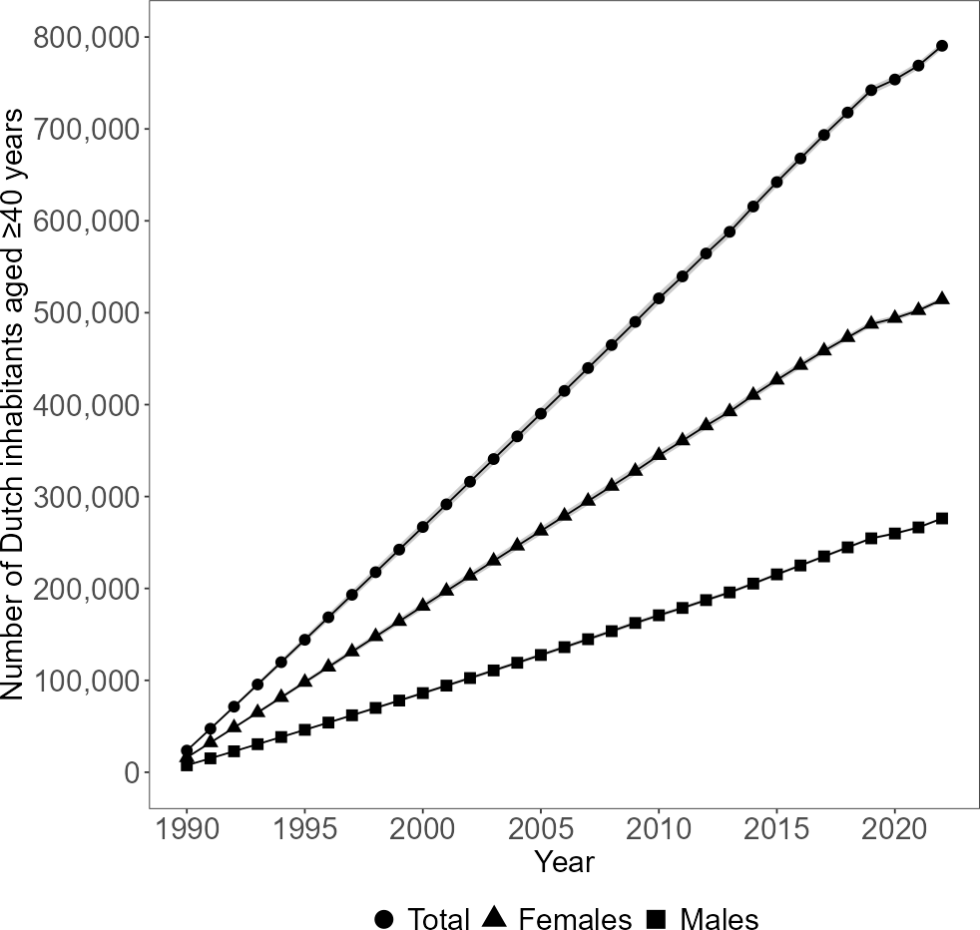
Total number of Dutch inhabitants aged ≥ 40 years living with at least one joint replacement over time, stratified by sex. The 95% CIs are small and therefore may not be visible.

**Fig. 4:**
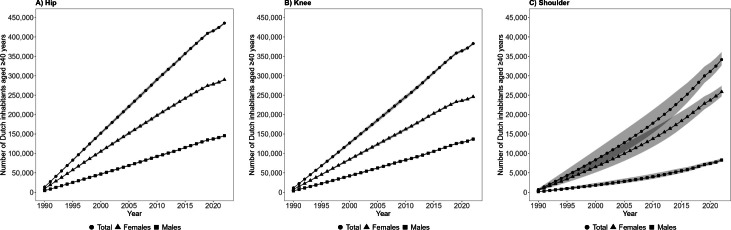
Total number of Dutch inhabitants aged ≥ 40 years living with at least one joint replacement over time, stratified by A) hip, B) knee, or C) shoulder replacement, and sex. The 95% CIs are small and therefore may not be visible.

In 2022, 791,000 (95% CI 787,000 to 794,000) people in the Netherlands were living with at least one joint replacement, representing 8.4% (95% CI 8.4 to 8.5) of the Dutch population aged ≥ 40 years (Figure 3; Supplementary Table i). Of these, 516,000 (65%) people were observed in the LROI. A total of 436,000 (95% CI 433,000 to 438,000) people were living with at least one hip replacement (Figure 4a), 383,000 (95% CI 380,000 to 386,000) people with at least one knee replacement (Figure 4b), and 34,000 (95% CI 33,000 to 36,000) people with at least one shoulder replacement (Figure 4c). This corresponds to 4.7% (95% CI 4.6 to 4.7), 4.1% (95% CI 4.1 to 4.1), and 0.4% (95% CI 0.3 to 0.4) of the Dutch population aged ≥ 40 years, respectively (Supplementary Table i). Among them, 289,000 (66%) people with at least one hip replacement, 246,000 (64%) people with at least one knee replacement, and 19,000 (57%) people with at least one shoulder replacement were observed from the LROI. The prevalence of people living with at least one joint replacement was 4.5% (95% CI 4.5 to 4.5) when considering the entire Dutch population of 17.6 million people. For hip, knee, and shoulder replacements, these were 2.5% (95% CI 2.5 to 2.5), 2.2% (95% CI 2.2 to 2.2), and 0.2% (95% CI 0.2 to 0.2), respectively.

The number of females having at least one hip (n = 290,000, 95% CI 288,000 to 292,000), knee (n = 246,000, 95% CI 244,000 to 248,000), or shoulder replacement (n = 26,000, 95% CI 25,000 to 27,000) in 2022 was 2.0, 1.8 and 3.1 times higher than the number of males with at least one hip (n = 146,000, 95% CI 145,000 to 147,000; Figure 4a), knee (n = 137,000, 95% CI 136,000 to 138,000; Figure 4b), or shoulder replacement (n = 8,300, 95% CI 7,900 to 8,800; Figure 4c), respectively. Females with at least one hip, knee, or shoulder replacement represent 6.0% (95% CI 6.0 to 6.1), 5.1% (95% CI 5.1 to 5.2), and 0.5% (95% CI 0.5 to 0.6) of the Dutch female population, respectively (Supplementary Table i). For males, this is 3.2% (95% CI 3.2 to 3.2), 3.0% (95% CI 3.0 to 3.0), and 0.2% (95% CI 0.2 to 0.2) of the Dutch male population, respectively.

**Fig. 5:**
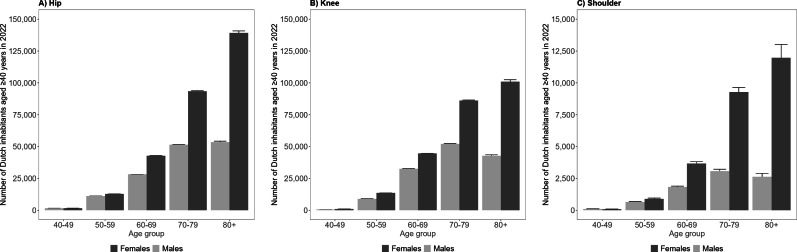
Total number of Dutch inhabitants aged ≥ 40 years living with at least one joint replacement in 2022, stratified by A) hip, B) knee, or C) shoulder replacement, age, and sex. The 95% CI are small and therefore may not be visible.

The most common age groups living with at least one joint replacement were the 70- to 79-year and ≥ 80-year age groups, representing 17% (95% CI 16 to 17) and 38% (95% CI 37 to 38) of the Dutch population aged 70 to 79 years and ≥ 80 years in 2022, respectively (Supplementary Table i). Among females, the highest number of women who had at least one hip, knee, or shoulder replacement was found in the ≥ 80-year age group, accounting for 27% (95% CI 27 to 27), 20% (95% CI 19 to 20), and 2.3% (95% CI 2.2 to 2.5) of the Dutch population aged ≥ 80 years, respectively (Figure 5; Supplementary Table i). In males, the highest number of men with at least one hip replacement was observed in the ≥ 80-year age group, while the highest number with at least one knee or shoulder replacement was observed in the 70- to 79-year age group (Figure 5). The highest prevalence for males was found in the ≥ 80-year age group for hip (16%, 95% CI 16 to 16), knee (13%, 95% CI 12 to 13), and shoulder (0.8%, 95% CI 0.7 to 0.8) replacements (Supplementary Table i). The number of people with at least one hip, knee, or shoulder replacement over time, stratified by age, is shown in Supplementary Figure a.

## Discussion

At the end of 2022, there were approximately 800,000 people living with a least one hip, knee, or shoulder replacement in the Netherlands. This corresponds to one in 12 Dutch inhabitants aged ≥ 40 years having at least one joint replacement. Among the ≥ 80-year-olds, approximately one in three Dutch inhabitants were living with at least one joint replacement. Females were more often living with at least one hip, knee, or shoulder arthroplasty compared to males due to the predominance of females in joint replacements, as well as their higher life expectancy.^15^

Both the estimated and observed incidences of hip and knee arthroplasties in this study are comparable to those reported by the Swedish Hip Arthroplasty Register and the Swedish Knee Arthroplasty Register, which have a long history of data collection.^16,17^ The annual incidence per 100,000 Swedish inhabitants aged ≥ 40 years was 225 in 1990 and 332 in 2010 for total hip arthroplasties, and 203 in 2005 and 268 in 2013 for total knee arthroplasties.^16,17^

Although comparing our findings with other countries may be complex due to differences in modelling methodologies, patient populations, and surgical indications, our findings appear to be in line with published prevalences estimated for all ages using data from Australia, Sweden, and the USA.^6,18,19^ In our study, 2.5%, 2.2%, and 0.2% of the entire Dutch population had at least one hip, knee, or shoulder replacement in 2022, respectively. In Australia, the prevalence of people with at least one hip, knee, or shoulder replacement was 3.4% in 2016.^6^ No distinction in the prevalences of different joints was reported. In Sweden, 3.3% of citizens were living with a hip or knee replacement at the end of 2022, of which 2.1% had at least one hip replacement and 1.5% had at least one knee replacement.^18^ In the USA, approximately 0.3% of the population had a shoulder replacement in 2017.^19^

The sum of the joint-specific proportions of Dutch people aged ≥ 40 years living with at least one hip, knee, or shoulder replacement was 9.2%, while 8.4% were living with at least one hip, knee, or shoulder replacement. This indicates that 9.5% of the proportion of people with at least one joint replacement have multiple primary joint replacements in different joints, either ipsilateral or cross-lateral. A previous LROI study on multiple primary hip and knee arthroplasties in osteoarthritic patients in the Netherlands found that 43% of the people underwent a second primary arthroplasty during their lifetime.^20^ The majority of these patients (83%) underwent a contralateral primary arthroplasty. Contralateral primary arthroplasties were not part of our study, as we used only the first hip, knee, or shoulder arthroplasty from patients to estimate the prevalence of people with at least one joint replacement.

Traditionally, hip arthroplasties have been more common than knee arthroplasties, as shown by both our and previous studies.^6,16,17^ This may be due to the earlier introduction of modern hip arthroplasty, earlier innovations in effective hip implants, and variations in the complexity of hip and knee arthroplasty procedures.^17,21,22^ However, previous studies also suggest that the number of primary knee arthroplasties will exceed the number of primary hip arthroplasties in the future as a result of a higher BMI, as well as increasing future incidences and prevalences of knee osteoarthritis.^6,16,17^ It remains unclear whether this trend also applies to the Dutch population.

Prevalence estimates of people living with at least one joint replacement may be important, as these estimates provide knowledge about the number of people who are at risk of complications following arthroplasty.^6^ These complications include PJIs, periprosthetic fractures, aseptic loosening, and dislocation, all of which may require reoperation or revision. Understanding the number of people living with a primary joint replacement can help project future healthcare procedures for these complications. Moreover, people with joint replacements require lifelong healthcare, including clinical and radiological follow-up. Therefore, the findings of this study may guide future research and assist healthcare providers, policy-makers, and researchers in understanding the impact of arthroplasty on the healthcare system, as well as in planning and allocating resources in both education and industry to meet the growing demand for arthroplasties, and to improve orthopaedic patient care and outcomes.

The findings of the present study should be interpreted carefully. No LROI data were available for hip and knee arthroplasties before 2007 and for shoulder arthroplasties before 2014. Additionally, completeness rates were suboptimal for hip and knee arthroplasties between 2007 and 2009 and for shoulder arthroplasties in 2014. Modelling historical incidences prior to LROI registration was essential, as there are still a considerable number of patients with non-registered hip, knee, or shoulder replacements. Of the current 800,000 people living with at least one joint replacement in the Netherlands, approximately 35% underwent their joint arthroplasty before the start of the LROI. Previous studies suggest the use of asymptotic regression models for predicting (future) incidences, which assume the incidence has an upper limit, rather than Poisson regression models, in which the modelled incidences could theoretically increase to infinity.^16,17^ However, we used Poisson regression models to estimate the incidences due to failures of the asymptotic regression models. We modelled historical incidences rather than predicting future incidences, making the theoretically unlimited incidence less relevant in our study.

Historical incidences were estimated since 1990. However, there may also still be patients alive in 2022 who received their joint replacement before 1990. While estimating historical incidences back to 1985 or 1980 increases the likelihood that patients who received their joint replacement before these years will not be alive in 2022, there is a risk of overestimating the historical incidences, as arthroplasties were performed only in small numbers in the Netherlands during the 1980s. Sensitivity analyses with the starting years 1985 and 1980 showed that by 2022, an estimated 805,000 and 812,000 people were living with at least one joint replacement, respectively. Published prevalence estimates from other countries were calculated from 1994 in Australia for hip, knee, and shoulder arthroplasties, from 1992 for hip arthroplasties and 1979 for knee arthroplasties in Sweden, and from 1988 in the USA for shoulder arthroplasties.^6,18,19^

Another limitation related to the estimated historical incidences may be changes in patient population and surgical indications over time. Nowadays, healthier and younger patients as well as more vulnerable elderly patients undergo hip, knee, or shoulder arthroplasties who may not have been eligible for these procedures in the past. Consequently, we may have slightly overestimated the number of people with at least one joint replacement in the Netherlands. Furthermore, all individuals aged ≥ 99 years were classified as 99-year-olds in this study, as CBS combined all Dutch people aged ≥ 99 years into one age category until 1995. Therefore, the survival of people aged > 99 years may be overestimated.

In contrast, while the LROI has achieved a relatively high completeness rate of over 90% for registered primary hip, knee, and shoulder arthroplasties in recent years, there have been cases of patients with non-registered hip, knee, or shoulder replacements.^1^ Therefore, this study may underestimate the number of people with at least one joint replacement in the Netherlands. Moreover, patients aged < 40 years were not included in this study, as the number of patients was too small to reliably estimate the prevalence for this group. In addition, we have not taken into account replacements of other joints, such as ankles, elbows, wrists, and fingers. However, the number of these arthroplasties is relatively low in the Netherlands.^1^ Nonetheless, LROI data, which offer a nationally representative sample of the Dutch population, are the best available data to estimate the number of people living with at least one joint replacement in the Netherlands.

In conclusion, by the end of 2022, approximately 800,000 people in the Netherlands were living with a least one hip, knee, or shoulder replacement, corresponding to one in 12 Dutch inhabitants aged 40 years or older. More females than males were living with at least one joint replacement. The proportion of the population with at least one joint replacement was highest among individuals aged 80 years or older. These findings may provide a better understanding of the burden of arthroplasty in the Netherlands.

**Take home message**

- The prevalence of people living with a joint replacement provides insight into the number of individuals at risk for complications associated with arthroplasty.

- This study is the first to estimate the prevalence of people living with at least one hip, knee, or shoulder replacement in the Netherlands and shows that a total of 8.4% of the Dutch population aged ≥ 40 years had at least one of these replacements in 2022.

## Data Availability

The datasets generated and analyzed in the current study are not publicly available due to data protection regulations. Access to data is limited to the researchers who have obtained permission for data processing. Further inquiries can be made to the Dutch Arthroplasty Register ().
